# Acute respiratory infection (COVID-19) risk prediction in travelers: A random forest model

**DOI:** 10.1016/j.idm.2025.12.021

**Published:** 2026-02-26

**Authors:** Jingbo Yu, Hao Yu, Yuming Wang, Qiang Zeng

**Affiliations:** a Orthopaedics Institute of Tianjin, Tianjin Hospital, Tianjin, People's Republic of China; b Institute of Operations and Quality Control, Tianjin Centers for Disease Control and Prevention, Tianjin, People's Republic of China; c School of Health and Management, Tianjin Medical College, Tianjin, People's Republic of China; d Institute of Occupational Health, Tianjin Centers for Disease Control and Prevention, Tianjin, People's Republic of China

**Keywords:** Prediction model, Severe acute respiratory infectious disease, International travelers, Travel history, Machine learning

## Abstract

**Background:**

Early screening during outbreaks of acute respiratory infections (ARIs) is critical for controlling disease spread among international travelers. However, the massive volume of traveler data generated in a short timeframe makes manual screening of suspected cases impractical for health quarantine officers. Prediction models for infection offer a promising solution to this challenge.

**Methods:**

Key predictive variables including travel history and seat numbers were extracted from passenger itineraries to construct the risk assessment model. Random forest algorithm and multivariate logistic regression were used to build prediction models of COVID-19 infection separately. Compare their performance through sensitivity(recall for the positive class), specificity, accuracy, AUC and Brier score. Sort the importance of variables through random forest algorithm.

**Results:**

The random forest model exhibited better discriminative ability and calibration. Variable importance analysis revealed travel history-derived factors as top predictors: close contacts(0.419), flight risk (0.286), and sojourn risk (0.182). Infection prevalence stratified by risk level: flight risk: low risk vs high risk: 0.7% vs 1.4%; sojourn risk: low risk vs high risk: 0.7% vs 2.0%; close contacts vs non-close contact: 0.3% vs 2.4%.

**Conclusions:**

The prediction model based on random forest algorithm has a better performance in identifying infected passengers than multivariate regression model. We should pay more attention on variables extracted by epidemiological history in building prediction model of respiratory infectious diseases. This model demonstrates strong potential for effectively responding to future outbreaks of acute infectious diseases such as COVID-19.

## 1 Introduction

Acute respiratory infectious diseases are characterized by distinct symptoms and rapid transmissibility. In a brief period, a large number of patients can emerge. Under certain circumstances, these diseases can even cross national and continental boundaries, giving rise to a global pandemic (Baker et al., 2022; Guan et al., 2004). Consequently, the demand for medical resources experiences a dramatic upsurge, thereby precipitating a medical exigency that exerts a deleterious impact on the global public health security landscape. Simultaneously, it has imposed significant constraints on people's routine activities and mobility patterns (Kim, 2020; Mao et al., 2020; Waterer, 2023).

Early-phase outbreaks of novel respiratory pathogens exhibit elevated case fatality rates, attributable to viral pathogenicity, immunological naivety as well as healthcare system strain. Between November 2002 and August 2003, the average mortality rate of Severe acute respiratory syndrome(SARS) cases reported by 29 countries is 9.3% (Chinese Center for Disease Control and Prevention). According to the World Health Organization(WHO), it was the first severe and readily transmissible new disease to emerge in the 21st century and showed a clear capacity to spread along the routes of international air travel (World Health Organization, 2003). Approximately 35% of Middle East Respiratory Syndrome(MERS) cases(firstly discovered in 2012) reported to WHO have died (World Health Organization, 2022). In March 2020, the World Health Organization announced that Corona Virus Disease 2019(COVID-19) had entered a pandemic state. As of the end of March 2020, the fatality rate of COVID-19 was 4.8%, according to data released by the WHO (World Health Organization, 2020).

The main factor that drives the spread of COVID-19 is closeness between an infected individual and a vulnerable individual. Close-contact gatherings frequently trigger superspreading events, characterized by disproportionate secondary transmission rates. The exponential growth of international air travel has become a critical accelerator of cross-border disease transmission (Hertzberg & Weiss, 2016; Mangili et al., 2015; Namilae et al., 2022; Shingleton et al., 2023). Modeling studies demonstrate an significant correlation between changes in the rate of spread and yearly fluctuations in monthly airline volume (Brownstein et al., 2006), with similar patterns observed in COVID-19 outbreaks (Chinazzi et al., 2020). Early detection of infected inbound travelers through airport screening reduces secondary transmission risk.

Epidemiological investigations consistently identify travel history and close contact exposure as significant predictors of infection risk during outbreaks, forming the basis of case definitions for diseases like MERS and COVID-19 (Duong & Waldman, 2016; Hon, 2013; Li et al., 2021).This study developed a predictive model for assessing COVID-19 infection risk among international arrivals by integrating traveler-specific data(detailed travel history (14-day country-specific residence), light itineraries (including aircraft seating configurations and transit duration) and epidemiological intelligence(WHO-reported country-level COVID-19 new cases), and investigated methodologies for quantifying travel-associated risk and delineating close contacts boundaries. We hope that this predictive model, when combined with antibody or viral nucleic acid screening methods, can classify the infection risk of inbound travelers, thereby slowing the rate of interpersonal virus transmission. Our study primarily consists of three components: 1) quantifying travel-associated risk and delineating close contacts boundaries, 2) establishing an infection prediction model, and 3) examining the epidemiological significance of key variables in the model.

## 2 Methods

### 2.1 Data collection and processing

We collected entry information from 175636 international travelers arriving at Tianjin's aviation port between March 2020 and July 2022. The collected information included: aircraft type, passenger seat number, flight origin, 14-day travel history, COVID-19 antibody test results, health declaration status, and requests for medical assistance upon arrival. Nine variables were extracted from the travelers' entry information, with detailed variable definitions provided in Table 1. Among these, the values of “flight risk” and “sojourn risk” were calculated based on the 14-day travel history and flight origin, combined with the WHO-published daily cumulative COVID-19 case data. The World Health Organization (WHO) updates daily new cases and cumulative cases for each country on a daily basis. We obtained the daily cumulative COVID-19 case data from WHO's official website and matched this information with the countries involved in passengers' 14-day travel history as well as the departure countries of their flights on the travel date.

**Table 1:** Definition and description of variables related to infection risk.

Variables	Definition
crew	Crew member or not
charter flight	Taking a chartered flight or not
sojourn number	Sum of countries traveled by passengers within 14 days.
referral	Refer to the hospital based on the abnormal health declaration results or medical assistance upon arrival
flight risk	Cumulative cases of COVID-19 at the departure place of the flight that day
sojourn risk	Cumulative cases of COVID-19 in the passenger's residence 14 days before departure
symptom COVID-19	Having symptoms(fever, cough, diarrhea etc.) related to COVID-19 or not
antibody test	The COVID-19 antibody test results of passengers at customs, including: IgM(−) IgG(−), IgM(−) IgG(+), IgM(+) IgG(−), IgM(+) IgG(+)
close contacts	Passengers in the same row, the first three rows, and the last three rows of seats for confirmed cases on the same flight, and all passengers on the same flight when the confirmed case is more than 15 will be defined as close contact

Calculation of sojourn risk: on the departure date, the sum of daily cumulative cases from all countries involved in the 14-day travel history constituted the sojourn risk value.

Calculation of flight risk: the COVID-19 cumulative case count in the departure country on the day of flight departure.

All collected data were standardized prior to analysis. Data processing and statistical analyses were performed using Python 3.10.3.

The dataset was randomly split into training and testing subsets at an 8:2 ratio. For model development, we independently implemented both random forest and logistic regression algorithms on the training set. Model predictive performance was subsequently evaluated using the held-out test set.

### 2.2 Definition

The detailed definitions of all variables are presented in Table 1.

### 2.3 Logistic regression-based predictive modeling

To mitigate potential multicollinearity issues that could compromise the predictive accuracy of our logistic regression model, we performed correlation analysis on all nine extracted variables. The analysis revealed a strong correlation between flight risk and sojourn risk (r = 0.974, *p* < 0.01; Table 2). Based on this finding, we retained sojourn risk for subsequent modeling due to its greater informational content while excluding flight risk to maintain model parsimony.

**Table 2:** Spearman correlation of variables related to infection risk.

	flight risk	sojourn risk	charter flight	close contacts	symptom COVID-19	crew	referral	antibody test	sojourn number
flight risk	1.000								
sojourn risk	0.974^a^	1.000							
charter flight	−0.239^a^	−0.243^a^	1.000						
close contacts	−0.064^a^	−0.056^a^	−0.023^a^	1.000					
symptom COVID-19	−0.012^a^	−0.013^a^	−0.005∗	0.006∗	1.000				
crew	0.106^a^	0.105^a^	−0.017^a^	−0.109^a^	−0.008^a^	1.000			
referral	−0.014^a^	−0.016^a^	−0.002	0.014^a^	0.345^a^	−0.019^a^	1.000		
antibody test	0.017^a^	0.008^a^	−0.009^a^	0.023^a^	0.097^a^	−0.012^a^	0.656^a^	1.000	
sojourn number	−0.024^a^	0.016^a^	−0.071^a^	0.045^a^	−0.002	−0.042^a^	0.016^a^	0.028^a^	1.000

a Correlation is significant at the 0.01 level (2-tailed). ∗. Correlation is significant at the 0.05 level (2-tailed).

A logistic regression model was implemented with a maximum of 10,000 iterations to ensure convergence, using the “liblinear” solver. Hyperparameter optimization was performed through grid search with stratified 5-fold cross-validation, evaluating regularization strength C (0.001, 0.01, 0.1, 1, 10, 100), penalty type (L1/L2), and class weighting (None/balanced). Model selection prioritized recall to optimize positive case identification. The optimal configuration utilized L1 penalty with C = 10 and balanced class weights, indicating moderate regularization with compensation for class imbalance. The best-performing parameter set identified by the grid search was then used to refit a final model on the entire training set.

### 2.4 Random forest-based predictive modeling

For the random forest model, hyperparameter tuning was performed using a stratified 5-fold cross-validation grid search. The parameter grid included n_estimators [100, 200], max_depth [None, 10, 20], min_samples_split (Guan et al., 2004; Mao et al., 2020), min_samples_leaf (Baker et al., 2022; Guan et al., 2004), and multiple class_weight options including 'balanced' and specific class weight ratios 0[0:1, 1:200] and [0:1, 1:250] to address class imbalance. The grid search employed recall as the optimization metric with stratified cross-validation, ultimately selecting the best parameter combination of n_estimators: 200, max_depth: 10, min_samples_split: 5, min_samples_leaf: 2, and class_weight: [0:1, 1:250] based on cross-validation performance. The best-performing parameter set identified by the grid search was then used to refit a final model on the entire training set.

### 2.5 Comparative analysis of logistic regression and random forest models

To comprehensively evaluate model performance, we assessed both discrimination and calibration capabilities. Discrimination was quantified using sensitivity(recall for the positive class), specificity, accuracy, Area under the ROC curve (AUC) and Brier score. Calibration was evaluated through Calibration plots. Inter-model agreement was analyzed by Spearman's rank correlation coefficient and Cohen's kappa coefficient.

Sensitivity(recall for the positive class): The proportion of actual positive cases correctly identified by the model, calculated as:

sensitivity=TP/(TP+FN)

where TP represents true positives and FN represents false negatives.

Specificity: The proportion of actual negative cases correctly identified by the model, calculated as:

specificity=TN/(TN+FP)

where TN represents true negatives and FP represents false positives.

Accuracy: The overall proportion of correctly classified instances, both positive and negative, calculated as:

Accuracy=(TP+TN)/(TP+TN+FP+FN)

AUC: A comprehensive measure of the model's discriminatory ability across all classification thresholds, ranging from 0.5 (no discrimination) to 1.0 (perfect discrimination).

Brier score: A proper scoring rule that measures the accuracy of probabilistic predictions, calculated as the mean squared difference between predicted probabilities and actual outcomes. A lower Brier score indicates a higher accuracy of the model's predictions, meaning that the model is closer to the actual results.

## 3 Results

### 3.1 Baseline characteristics of the study cohort

**Fig. 1:**
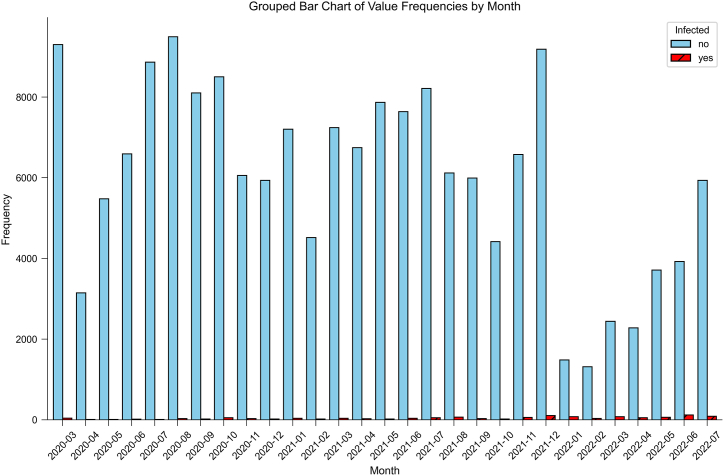
Infection Status among Arrival Passengers: March 2020 to July 2022 Blue bars: Non-infected passengers; Red bars: Infected cases.

**Fig. 2:**
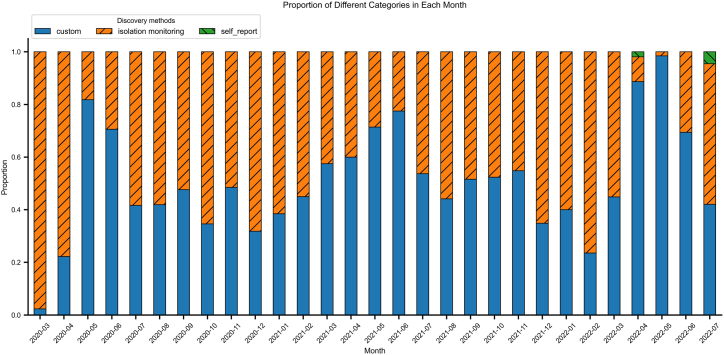
Monthly Distribution of Infection Discovery Methods among Arrival Cases Blue: Detected at border screening; Orange: Found during quarantine; Green: Self-declaration of abnormal health status before entry.

Between March 2020 to July 2022, A total of 175636 people entered the country through Tianjin air ports, with 1305 people infected. Numbers of inbound passengers and inbound infected individuals were shown in Fig. 1. There are three ways to screen infected passengers among inbound passengers: self-declaration of abnormal health status before entry; virus nucleic acid testing(+) at customs; virus nucleic acid testing(+) during isolation monitoring. 780 passengers were confirmed through health declaration and customs nucleic acid testing, while 565 passengers were found during subsequent isolation monitoring. Proportion of each way in every month was shown in Fig. 2. Most of them were discovered through customs inspection and isolation monitoring, and the proportion of s self-declaration is very low. 38744 individuals were excluded because of missing information about seat number and 14 days of travel history. A total of 136891 objects were included for subsequent modeling, and 950 individuals were positive, with a final event incidence rate of 0.69%.

### 3.2 Performance of the two models

The sensitivity(recall for the positive class), specificity, accuracy of the random forest model in the training set are 0.90, 0.74, 0.74; in the test set are 0.80, 0.74, 0.74. The sensitivity(recall for the positive class), specificity, accuracy of the logistic regression model in the training set are 0.72, 0.79, 0.79; in the test set are 0.78, 0.78, 0.78. The confusion matrixes of two models are shown in Table 3.

**Table 3:** Confusion matrixes of models.

		Random Forest			Logistic Regression		
			Predict			Predict	
Train set			-	+		-	+
	Actual	-	80405	28344	-	85472	23277
		+	77	686	+	217	546
Test set			-	+		-	+
	Actual	-	20104	7088	-	21332	5860
		+	38	149	+	42	145

To evaluate the generalization capability and stability of the constructed models, both logistic regression and random forest classifiers underwent stratified five-fold cross-validation. Performance metrics for each fold of the two models are detailed in Table 4. The logistic regression model achieved a mean recall(weighted) of 0.789(±0.004), while the random forest model attained a mean recall(weighted) of 0.758(±0.015), indicating consistent and stable classification performance across different data splits for both algorithms. The small standard deviations further support the strong robustness and generalizability of both models.

**Table 4:** 5-fold cross-validation results for Random Forest and Logistic Regression models.

	Fold	Accuracy	Precision (weighted)	Recall (weighted)	F1 (weighted)	AUC
Random Forest	Fold1	0.73	0.99	0.73	0.84	0.85
	Fold2	0.76	0.99	0.76	0.86	0.88
	Fold3	0.76	0.99	0.76	0.86	0.85
	Fold4	0.76	0.99	0.76	0.86	0.85
	Fold5	0.78	0.99	0.78	0.87	0.86
	Mean ± SD	0.76 ± 0.01	0.99 ± 0	0.76 ± 0.01	0.86 ± 0	0.86 ± 0.01
Logistic Regression	Fold1	0.79	0.99	0.79	0.88	0.81
	Fold2	0.79	0.99	0.79	0.87	0.84
	Fold3	0.79	0.99	0.79	0.88	0.82
	Fold4	0.79	0.99	0.79	0.88	0.80
	Fold5	0.78	0.99	0.78	0.87	0.79
	Mean ± SD	0.79 ± 0	0.99 ± 0	0.79 ± 0	0.88 ± 0	0.81 ± 0.01

Precision (weighted): The average of per-class precision scores, weighted by class sample size; Recall (weighted): The average of per-class recall scores, weighted by class sample size; F1 (weighted): The harmonic mean of the weighted precision and weighted recall scores.

**Fig. 3:**
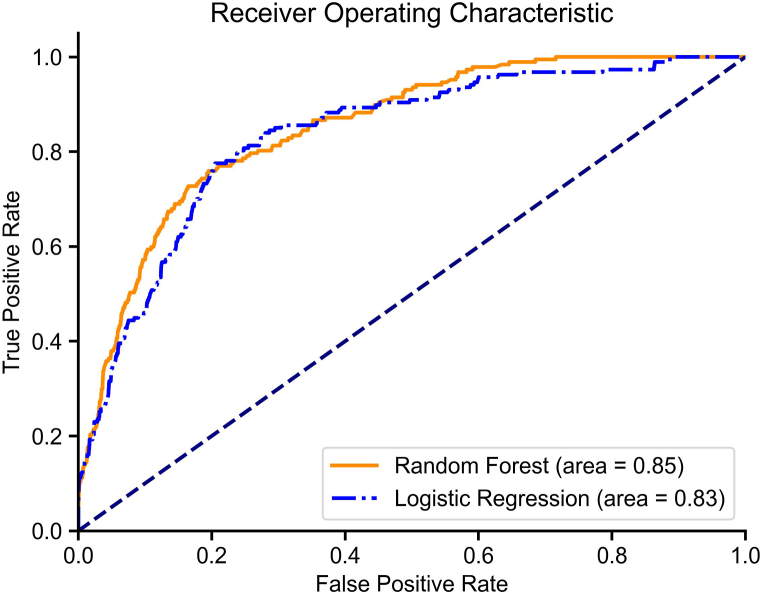
Model Performance Evaluation: Random Forest and Logistic Regression Dashed line: Random classifier; Orange line(solid): Random Forest(AUC = 0.85); Blue line(dashdot): Logistic Regression(AUC = 0.83).

**Discrimination:** The two models exhibited comparable performance based on the Brier score, with only a marginal difference between them (Random Forest: 0.18; Logistic Regression: 0.17).The model built by the random forest algorithm performs better in AUC(random forest, 0.85; logistic regression, 0.83). Fig. 3 depicts differences in AUC between the two models.

**Fig. 4:**
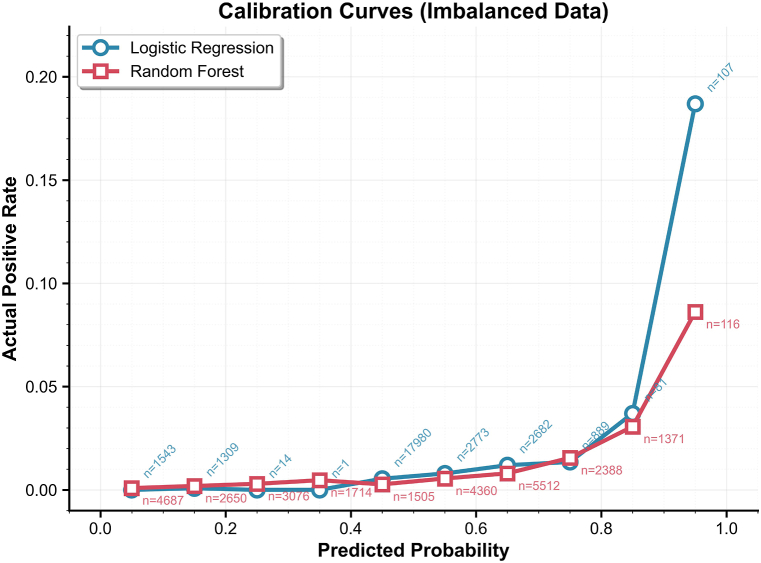
Calibration Curve Comparison: Random Forest vs Logistic Regression Blue: Logistic Regression; Orange: Random Forest; n represents the number of predicted infections in each bin.

**Calibration:**Calibration plots were used to quantified the calibration performance of the two models. The calibration curves of both models are positioned closer to the x-axis, indicating that the observed positive rate is consistently lower than the predicted probability across all bins. This suggests that both models exhibit systematic overestimation of the risk. Overall, the upward trend of the curves demonstrates that both models possess good discriminatory ability in ranking the infection risk among passengers. Specifically, the calibration curve of the logistic regression model shows greater fluctuation, whereas that of the random forest model is smoother and more stable (see Fig. 4).

**Agreement:** The predicted probabilities from the two predictive models demonstrated a strong correlation (Spearman's ρ = 0.744, *p* < 0.001) and reached a moderate level of agreement in their final classification outcomes (Kappa = 0.574).

### 3.3 Explanation of variables importance in models

**Fig. 5:**
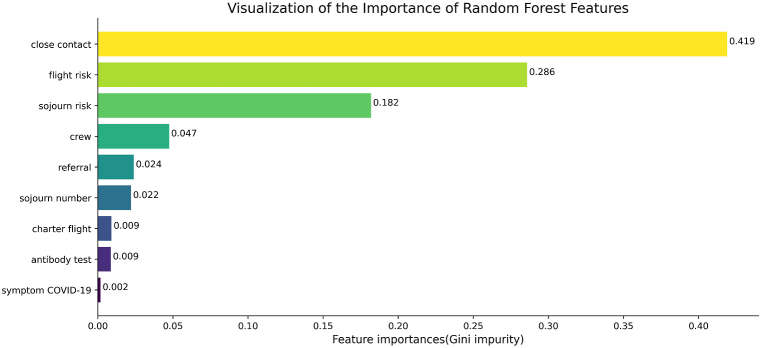
Feature Importance in Random Forest Model Higher values indicate stronger predictive power.

**Random forest:** The relative importance of predictive features was quantified using the Gini importance method inherent to the random forest algorithm. The ranked importance scores were shown in Fig. 5. Three primary determinants of infection risk were identified by the random forest algorithm based on normalized Gini importance: (1) close contacts (importance: 0.419), indicating exposure to confirmed cases during travel, (2) flight risk(importance: 0.286), representing COVID-19 prevalence in the departure region, and (3) sojourn risk(importance: 0.182), reflecting cumulative exposure in visited locations.

**Fig. 6:**
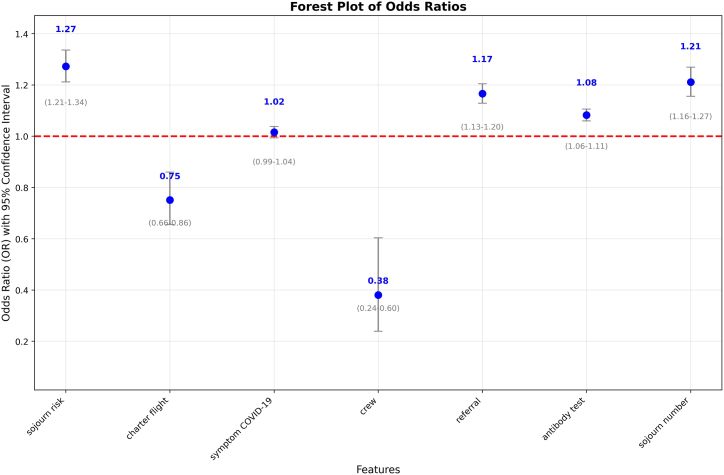
Odds Ratios with 95% Confidence Intervals: Logistic Regression Results All figures have been designed for monochrome (black and white) printing. Color differences in the original figures are represented by different hatch styles and line styles.

**Logistic regression:** The logistic regression model identified several significant predictors of COVID-19 infection risk (Fig. 6): Protective Factors: crew status (OR = 0.46, 95% CI:0.29-0.73), charter flight (OR = 0.77, 95% CI: 0.67-0.88); Risk Factors: close contacts (OR = 2.39, 95% CI: 2.25-2.53), referral (OR = 1.18, 95% CI: 1.14-1.22), positive antibody test (OR = 1.07, 95% CI: 1.04-1.09), sojourn risk (OR = 1.22, 95% CI: 1.17-1.28), multiple sojourn countries (OR = 1.16, 95% CI: 1.11-1.22). Close contacts showed the strongest risk elevation (139% increase vs non-contacts).

## 4 Discussion

During the COVID-19 pandemic, universal nucleic acid testing and quarantine monitoring were implemented for all inbound travelers as primary containment measures. However, our data reveals that cases detected at entry (Fig. 2) represented only a fraction of ultimately confirmed infections, indicating that sole reliance on arrival testing fails to intercept all infected individuals. This strategy alone showed limited effectiveness in comprehensive case detection. This detection gap leaves destination communities vulnerable to ongoing transmission risks from undetected cases. The implementation of universal quarantine for all arrivals presents substantial practical challenges, including excessive allocation of public health resources, significant economic burdens, and unintended consequences for essential international travel and cooperation. Our study advocates a risk-stratified approach to identify high risk inbound travelers, enabling targeted interventions (e.g., prioritized monitoring and travel advisories) while preserving mobility for low risk populations to mitigate local transmission risks. This precision public health strategy balances epidemic control with sustainable international connectivity, addressing the critical need for more efficient border health measures during prolonged pandemic response. The prediction model constructed in this study and the key risk variables identified can support the prevention and control of similar acute respiratory infectious diseases in the future, serving as an effective supplementary tool for pre-entry screening at ports of entry.

While predictive models for infectious disease epidemics exist in the literature (Gu et al., 2021; Kim & Ahn, 2021; Lee et al., 2021; Wu et al., 2022; Zhou et al., 2022), few studies have focused on individual-level prediction of acute respiratory infection risk. Wang et al. simulated the respiratory activities of individuals infected with COVID-19 within an aircraft cabin, determining the infection status of passengers by calculating the number of COVID-19 viral copies inhaled by each individual (Wang et al., 2023). By integrating passengers' travel histories and flight information, we developed a predictive model to identify high risk individuals among inbound travelers.

The random forest algorithm has been widely applied in medical research (Cardon et al., 2022; Xin & Ren, 2022; Yu et al., 2024). We used the random forest algorithm and logistic regression to build prediction model separately, and assessed the stability of their performance through five-fold cross-validation. The prediction model based on Random Forest demonstrated better performance in predicting positive cases (higher sensitivity) and exhibited greater stability in the calibration curve. To further elucidate the predictive performance of our random forest-based model, we dichotomized all inbound passengers in the test set into high risk (predicted probability ≥0.5) and low risk (predicted probability <0.5) groups based on the model's predicted probabilities. The number of actually infected individuals was higher in the high risk group compared to the low risk group (low vs high: 0.19% vs 2.06%). Based on the classification results of the random forest prediction model (confusion matrix of test set), the sensitivity was 80%, the positive predictive value was 2.06%, and the proportion of predicted positive cases among the total population was 26.43% (7237/27379). Compared with comprehensively quarantining and monitoring all inbound passengers, the strategy of "entry screening combined with a passenger risk classification model" substantially reduces the number of people requiring isolation. Although this approach may miss some infections, leading to a modestly lower detection rate, it still reduces the risk of imported cases causing community transmission more effectively than symptom screening without quarantine. A study has indicated that a higher number of imported cases increases the probability of a large outbreak, assuming SARS-like superspreading events can occur (Kucharski et al., 2020).

Identifying suspected infected individuals by travel history or close contacts are supported by literatures (Hertzberg et al., 2018; Liu et al., 2022; Paquet et al., 2022). Accurately inquiring and collecting travel history is helpful for doctors diagnosing (Burchard, 2014). A study in USA tried to extract travel history from clinical notes automatically for informing the detection of emergent infectious disease events, and it did produce an effect in early stage (Peterson et al., 2021). In our research, travel history also been effective in the model. The variables relevant to travel history(flight risk, sojourn risk)were identified as the second and third most influential variables for predicting. Exactly as we supposed, cumulative cases can serve as a quantitative indicator of the risk of travel history. In our study, the difference is significant in the proportion of actual infected individuals in different risk level of travel history (flight risk: low risk vs high risk: 0.7% vs1.4%; sojourn risk: low risk vs high risk: 0.7% vs 2.0%).

In the random forest model variable importance ranking, "close contacts" was identified as the most significant predictor. The concept of close contacts is related to people or animals living together ever (Li et al., 2020; Ploemacher et al., 2020; Shi et al., 2020). There are also studies that define it as related to transportation vehicles (Liu et al., 2022). Although advanced air handling systems on modern aircraft were considered to limit the spread of viruses in aircraft environment, there are cases infected through air travel (Edelson, 2012). Studies have found that many serious infectious diseases are transmitted through air travel (Berruga-Fernández et al., 2021; Božič & Kanduč, 2021).

Regarding the basis for our classification, we referred to previous research on SARS, influenza and SARS-CoV-2. For instance, one study suggested that passengers seated within two rows of an infected individual had an approximately 6% risk of infection (Hertzberg & Weiss, 2016). A 2020 study investigating the in-flight transmission of SARS-CoV-2 surveyed 217 passengers on a commercial flight. The results revealed that 12 (75%) of the infected individuals were business class passengers, the same section where the only symptomatic infected person was seated (attack rate 62%). The study concluded that the large cluster of infections during this long-haul flight was most likely caused by in-flight transmission originating from a single symptomatic passenger. Seating proximity was significantly associated with an increased risk of infection (risk ratio 7.3, 95% CI 1.2–46.2) (Khanh et al., 2020). A German study defined close contacts as persons who had been within 2 m of an index case for more than 15 min, including all passengers seated within two seats of the index case, all crew members, and other individuals who had close contact with the index case. The final results revealed that out of all contact-traced cases(891), five suspected instances of in-flight transmission were identified (Pavli et al., 2020). Based on the findings in the literature and comprehensive considerations, in this study, the definition of close contacts was conservatively expanded to include passengers within 3 rows of an infected individual. To further enhance risk coverage, all passengers on flights with over 15 confirmed cases were also classified as close contacts. Our team analyzed confirmed cases from inbound flights during the study period and found that over half of the flights(59.26%) had no cases, and the vast majority(86.92%) had 0-3 positive cases. Only 8 flights reported more than 15 confirmed cases. Given that 15 represents the 99th percentile of case distribution in this cohort, we used it as a critical threshold. Once this threshold was exceeded, all passengers on such flights were considered to be at elevated exposure risk. During a pandemic, epidemiological resources are often extremely limited. Conducting precise close-contact determinations (including detailed tracing of movements, restroom usage, and close interactions) for international flights carrying hundreds of passengers requires substantial manpower and time. Such efforts may lead to delayed responses and potentially exacerbate the spread of the outbreak. The shift from time-consuming individual tracing to a rapid, conservative strategy based on group exposure risk represents a pragmatic approach to outbreak containment. We acknowledge that the current strategy for defining close contacts is relatively conservative, with its primary aim being to maximize the identification of potential infections in order to effectively prevent the importation and subsequent spread of the disease. We noted that the existing study (Wang et al., 2023) have assessed infection risk by simulating the respiratory activities of infected individuals of COVID-19 in aircraft cabins and calculating the number of viral copies inhaled by each passenger. Incorporating such respiratory activity simulations into our close contact criteria could potentially enhance the precision of our approach. This study has certain limitations in the refinement of exposure risk assessment of close contact. In our study, the number of actually infected individuals in the close contact group was 8 times higher than that in the non-close contact group.

While COVID-19 symptoms (Baj et al., 2020) and antibody test results (Bortz et al., 2021) represent direct biological markers of infection, their relatively low predictive rankings (ranked 9th and 8th, respectively) may reflect several operational and epidemiological factors: (1) Asymptomatic presentation; (2) Non-specific symptom manifestation during early infection; (3) Limited symptom reporting without active prompting; (4) Incubation period dynamics; (5) In-flight acquisition; (6) Temporal lag between infection and antibody seroconversion. This suggests that while biologically plausible, symptom-based screening and antibody testing demonstrate limited utility for early identification in travel settings compared to epidemiological predictors like travel history.

Findings from this study suggest that key variables extracted from passenger entry and flight information(such as flight risk, sojourn risk, and close contacts) can be extended to the screening of acute respiratory infectious diseases with similar transmission characteristics, thereby providing a reference for targeted prevention and control at ports of entry.

Admittedly, the limitations of this study should be acknowledged. Firstly, the study is limited by insufficient diversity of variables. Our variable selection was constrained by the limitations inherent to border screening documentation: (1) Restricted to administratively collected entry records; (2)Contained incomplete fields; (3) Self-reported travel histories susceptible to recall bias; (4) Symptom reporting dependent on passive declaration. Secondly, after comprehensive consideration, we consciously avoided further optimizing the class imbalance parameters in the Random Forest model to pursue higher sensitivity, as doing so would substantially increase the proportion of predicted positive cases. This would result in a larger number of travelers being classified into the high risk category, potentially causing unnecessary concern among those flagged as positive and raising operational management costs. Therefore, striking a balance between achieving high recall for positive cases and controlling the rate of false positives remains a critical challenge for practical implementation in the future.

The advent of big data analytics and advanced computational methods has transformed our capacity to extract meaningful insights from complex datasets. This study builds a more effective tool by absorbing the essence of the past method and combining it with new technique, demonstrating how technological innovation can complement rather than replace validated traditional approaches.

## 5 Conclusion

Our study establishes a predictive model for acute respiratory infections (including COVID-19) among international air travelers, integrating epidemiological risk factors with machine learning to enable early identification of high-risk individuals at entry points. Meanwhile, our study validates the significance of epidemiology information(travel history and close contacts) in travel related infection risk assessment. Globally, with the advance of transportation vehicles, infectious diseases with clear or unclear pathogens spread across national or even continental borders. In this context, the variable definitions related to travel history and the contact tracing strategy developed in this study can be effectively applied to screening inbound passengers for risks associated with acute respiratory infections.

## CRediT authorship contribution statement

**Jingbo Yu:** Writing – review & editing, Writing – original draft, Visualization, Validation, Supervision, Software, Methodology, Investigation, Formal analysis, Data curation, Conceptualization. **Hao Yu:** Writing – review & editing, Supervision, Investigation, Data curation, Conceptualization. **Yuming Wang:** Writing – review & editing, Writing – original draft, Investigation, Data curation. **Qiang Zeng:** Writing – review & editing, Supervision, Project administration, Investigation, Conceptualization.

## Ethics approval and consent to participate

The Biomedical Ethics Committee of Tianjin Center for Disease Control and Prevention approved the research and clearly stated that the need for consent to participate was waived. The study was conducted in accordance with approved guidelines and followed the Declaration of Helsinki. Trial registration number:TJCDC-R-2022-032.

## Availability of data

The data underlying this article cannot be shared publicly due to privacy reason. The part of data(not involving private information or hiding private information) will be shared on reasonable request to the corresponding author.

## Consent for publication

Not applicable.

## Funding

This work was supported by Major Science and Technology on Public Health of Tianjin [grant numbers 21ZXGWSY00010], Tianjin Key Medical Discipline (Specialty) Construction Project [grant numbers TJYXZDXK-066B], Tianjin Health Commission Science and Technology Program [grant numbers TJWJ2022MS046], and Tianjin Key Medical Discipline Construction (TJYXZDXK-3-020B).

## Conflict of interest

The authors have declared no conflicts of interest.
